# A Rare Case of Pregnancy Complicated by Uterine Prolapse and Cervical Fibroid

**DOI:** 10.7759/cureus.9026

**Published:** 2020-07-06

**Authors:** Archana Barik, Alokananda Ray

**Affiliations:** 1 Obstetrics and Gynaecology, Tata Main Hospital, Jamshedpur, IND

**Keywords:** uterine prolapse, cervical, fibroid, pregnancy

## Abstract

Uterine prolapse and cervical fibroid are two common gynecological conditions but relatively uncommon in pregnant women. However, these conditions can complicate normal pregnancy by significantly altering its course and outcome. Obstetricians should, therefore, familiarize themselves with the presentations, challenges, and outcomes of these unique situations to effectively manage the patient in the perinatal period. We report a rare case, where both uterine prolapse and cervical fibroid coexisted in a single pregnancy.

## Introduction

Uterine prolapse during pregnancy is rare with an incidence of one in 10000-15000 pregnancies [1]. This number has reduced further worldwide over the past few decades, probably due to a decrease in parity. However, in developing countries, the number is still high because of malnutrition, persistence of multiparty, and decreased interval between consecutive pregnancies [2].

Similarly, the coexistence of uterine leiomyoma in pregnancy is uncommon with prevalence estimated to be 1.6%-10.7% [3-4]. Amongst them, cervical fibroid is the rarest accounting for less than 1% [5].

Both uterine prolapse and cervical fibroid can have a significant impact on the progression and outcome of pregnancy. Some of the reported adverse events are retention of urine, infection, septicemia, preterm labor, obstructed labor, antepartum and postpartum hemorrhage, increased chance of operative delivery, and even maternal death [6-7].

It is highly unusual to find both these clinical entities coexisting in a single patient. We report a woman who presented in active labor with stage 3 uterine prolapse and later on examination she was found to have a cervical fibroid as well. She went into obstructed labor and a cesarean section was performed to deliver the fetus.

## Case presentation

A 33-year-old woman, gravida 4, para 3, live 3 at 35 weeks 4 days of pregnancy presented to the labor room with pain in abdomen for two days and something coming out of the vagina for one month. There was a history of difficulty in passing urine and stool for one month. All her previous deliveries were vaginal and in short intervals. There was no history of uterine prolapse in her previous pregnancies. She also had undergone right ovarian cystectomy one year back.

**Figure 1:**
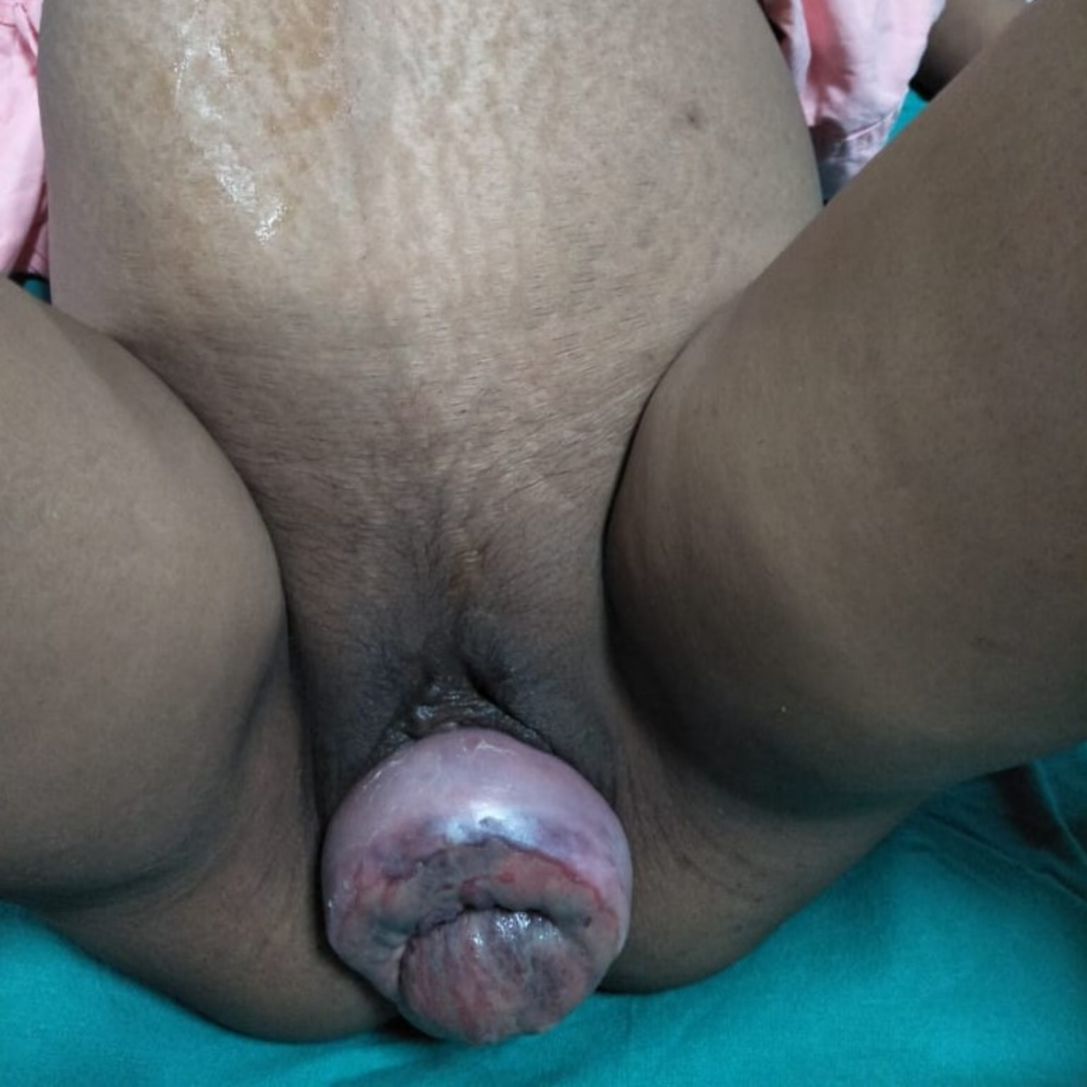
Edematous prolapsed cervix.

On abdominal examination, the uterus was found to be 34-36 weeks of size with mild contraction. The fetus was in the longitudinal lie with cephalic presentation and fetal heart sound was audible. On pelvic examination, stage 3 uterine prolapse with edematous cervix and parous size os was found (Figure 1). Pelvic organ prolapse quantification (POPQ) was done and it revealed point C as the leading edge with other measurements being Aa-2, Ap-2, Ba-2, Bp-2, C+3, D-1, gh 6, pb 2, tvl 6. The reposition of the prolapsed uterus was tried but could not be achieved.

**Figure 2:**
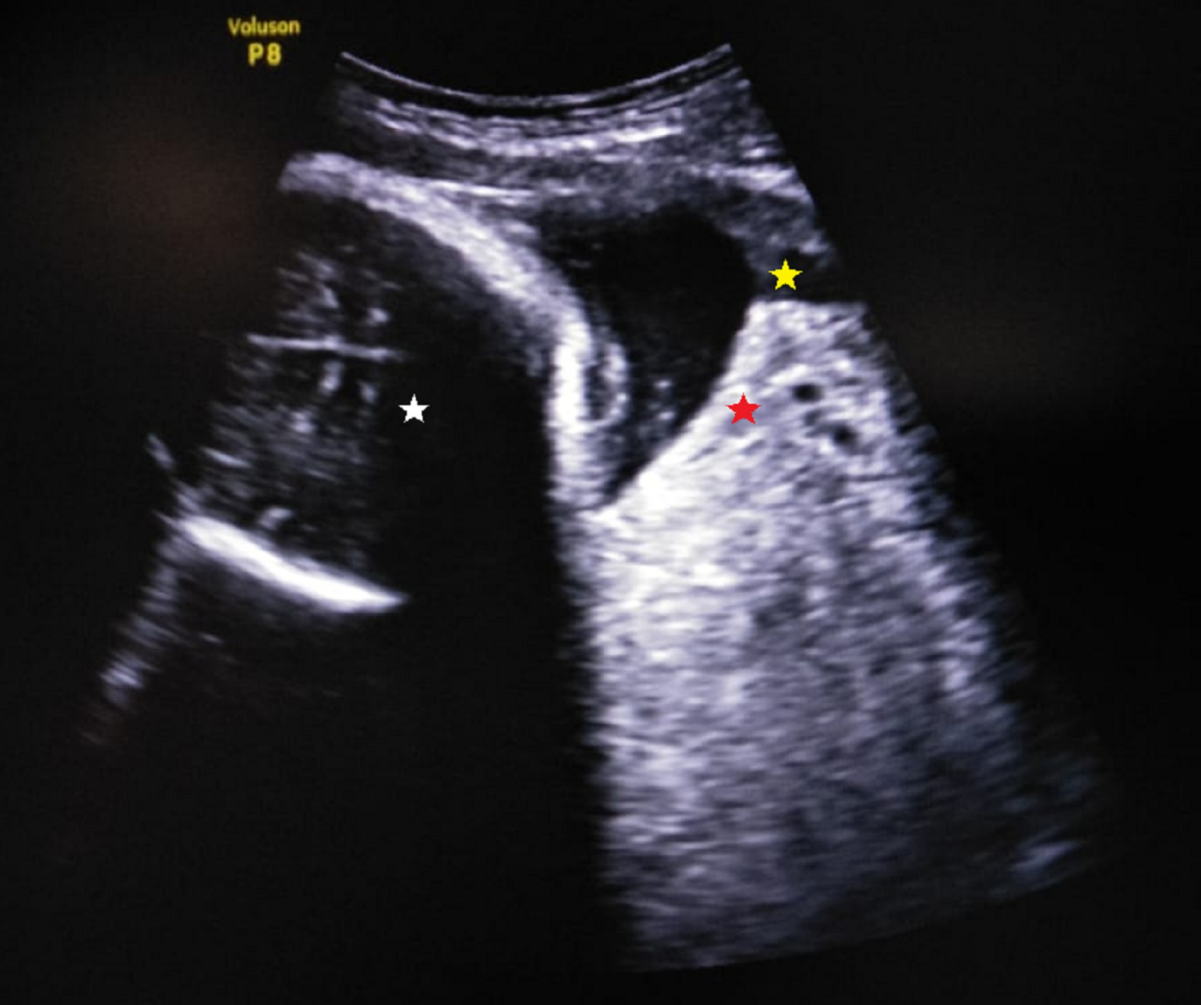
Ultrasound of pelvis. Ultrasound image showing cervical fibroid (red star) of size 13 cm x 10 cm arising from the posterior cervix and bulging into the cervical canal (yellow star). The fetal head (white star) can be seen above the fibroid mass.

Ultrasound examination revealed a single live fetus with gestational age corresponding to 34 weeks and placenta lying in the anterior and upper segment of the uterus. A homogeneous mass of size 13 cm x 10 cm was seen arising from the posterior part of the cervix lying below the fetal head (Figure 2).

Cesarean section was planned to deliver the fetus as the patient was progressing into obstructed labor. Preoperatively blood was cross-matched anticipating massive postpartum hemorrhage. Pfennaensteil abdominal incision was given and the uterine incision was put transversely avoiding the fibroid in the incision line during surgery. A cervical fibroid of size 10 cm x 10 cm partially covering the os was detected. A healthy child was delivered by vertex with a good APGAR score. The intraoperative blood loss was average in amount and we did not encounter any complications during surgery. Postoperatively, the prolapsed vaginal mass was successfully reposed and the rest of her hospital stay was uneventful. The patient was discharged with advice for further monitoring and management of the cervical fibroid and uterine prolapse in the postpartum period but she was lost to follow up.

## Discussion

Uterine prolapse either exists before or has an acute onset during pregnancy. Pre-existing prolapse is relatively uncommon and usually resolves as the uterus becomes an abdominal organ in the latter part of the pregnancy [6]. Whereas, acute onset prolapse developing during pregnancy is more common, usually reported in the third trimester [8]. The etiology in the latter is presumed to be due to frail pelvic support, caused by repeated trauma to the pelvic floor or congenital connective tissue disorder [9]. Besides that high serum level of cortisol and progesterone during pregnancy softens and stretches the pelvic musculature and weakens the pelvic support leading to prolapse [10].

Pregnancy with uterine prolapse categorizes a woman as high risk because of impending complications in the peripartum period. The common complications are patient discomfort, cervical desiccation, ulceration, urinary tract infection, acute retention of urine, preterm labor, and abortion. In neglected cases, the prolapsed mass may become edematous and incarcerated leading to cervical dystocia and obstructed labor culminating in uterine rupture if the emergency cesarean section is not performed [11]. Hence, early recognition and treatment of prolapse are important in the antenatal period to prevent complications. Conservative management in the form of bed rest, Trendelenburg position with manual reposition of the prolapsed mass is recommended. A pessary can be used to manage the prolapse throughout the pregnancy until the onset of labor [12-13]. Laparoscopic colposuspension surgery in early pregnancy has been reported, but it should be considered only when conservative management fails [14].

Operative delivery is usually the norm, especially in the cases of severe prolapse. It is proven that the cesarean section is protective against the persistence of prolapse after delivery [6]. However, vaginal delivery is possible and in some cases, Dührssen incision in the cervix is used to facilitate it [2].

Fibroids in pregnancy are increasingly becoming more common nowadays due to delay in childbearing. However, the cervical fibroid is extremely rare and accounts for less than 1% of all fibroids in pregnancy and the literature data are limited in describing its management [5]. Nevertheless, it can significantly affect the progression of pregnancy and its outcome. Some of the known complications are urinary retention due to pressure effects on the bladder or urethra, degeneration of fibroid, and preterm labor. During labor, it may induce malpresentation, dystocia, obstructed labor and there may be increased hemorrhage during delivery [7].

There are several types of cervical fibroid depending upon its origin from the supravaginal or vaginal portion of the cervix with varied clinical presentations. Supravaginal fibroids can surround the entire cervical canal and may lie centrally in the pelvis [15]. Obstructed labor is one of the major concerns in this scenario and the usual mode of delivery is by cesarean section [16]. Moreover, as the fibroid grows in size, it displaces the lower segment of uterus high up, and during surgery vertical midline abdominal incision may be needed [15]. There is always a higher risk of massive intraoperative hemorrhage needing emergency hysterectomy and hence, advanced preoperative preparation is mandatory to prevent complications.

Myomectomy during cesarean section is controversial, as there is a risk of profuse hemorrhage. Post-delivery conservative management should be considered as the fibroid usually degenerates by spontaneous thrombosis of its feeding blood vessels. However, other management options like uterine artery embolization, myomectomy, and hysterectomy can be employed post-delivery considering the patient’s symptoms, fertility desire, and site of the mass [15].

The uncommon presentation of our case initially created a diagnostic dilemma. Though the uterine prolapse was obvious, the diagnosis of cervical fibroid was only made after the ultrasound examination.

The etiology of prolapse might be due to weak pelvic support caused by repeated previous pregnancies with short interval time or might be because of the hormonal changes during pregnancy as there was no prior history of any connective tissue disorder affecting the pelvic musculature. The prolapsed mass could not be reposed back as it was grossly edematous and also the centrally placed cervical fibroid prevented us from doing so. The fibroid was also plausibly impinging on the bladder base causing urinary symptoms in the antenatal period. It was originating from the supravaginal portion of the posterior cervix obliterating the cervical canal, and hindering the descent of the fetal head, and hence cesarean section was performed for delivery. Intraoperatively, the uterine incision was carefully given to avoid the fibroid in the incision line and by doing so excessive intraoperative bleeding was prevented. She was planned for laparoscopic sacrocolpopexy for uterine prolapse later along with conservative management for fibroid. However, for symptomatic cervical fibroid, a hysterectomy would have been the management of choice in our case.

## Conclusions

Pregnancies complicated with pelvic organ prolapse and cervical fibroid are rare. Obstetricians have limited exposure in managing such unique situations. It is thus necessary to be aware of the complications and challenges expected in these scenarios to ensure safe pregnancy and delivery.
